# Acupuncture and herbal moxibustion for the treatment of ‘BiQiu’ (allergic rhinitis symptoms) in a Hong Kong Chinese medicine clinic: a randomized controlled trial

**DOI:** 10.1186/s13020-019-0272-7

**Published:** 2019-11-08

**Authors:** Ting Yiu Yung, Hongwei Zhang, Lap Che Tang, Lang Zhang, Chak On Law, Wai Man Tam, Chun Wai Chan, Heng Chun Chen, Man Hork Lee, Tat Chi Ziea, Fung Leung Ng, Zhi Xiu Lin

**Affiliations:** 1 Nethersole Chinese Medicine Service cum The Chinese University of Hong Kong Chinese Medicine Clinical Training and Research Centre, Hong Kong, China; 2 School of Chinese Medicine, The Chinese University of Hong Kong, Shatin, N.T, Hong Kong, China; 3 Chinese Medicine Department, Hospital Authority, Hong Kong, China

**Keywords:** Acupuncture, Allergic rhinitis, Biqiu, Randomized Clinical Trial, Herbal moxibustion, Herbal paste

## Abstract

**Background:**

Allergic rhinitis (AR) is a common disease. No evidence is available for the clinical application of acupuncture and moxibustion for the management of AR symptoms in Hong Kong. This study aimed to evaluate the clinical effectiveness of acupuncture with or without herbal moxibustion on relieving AR symptoms in the Hong Kong population.

**Methods:**

A single-centre, randomized, assessor-blinded, controlled trial with three parallel arms (acupuncture alone, acupuncture combined with herbal moxibustion treatment and waitlist) was designed. Groups with acupuncture treatment received treatment 3 times per week for a total of 12 sessions in 4 weeks. Acupuncture combined with herbal moxibustion treatment group received herbal moxibustion once per week for a total of 4 sessions over 4 weeks in addition to acupuncture treatment. Participants in the waitlist group received no treatment. All patients received advice on healthy lifestyle, diet, and exercise.

**Results:**

Ninety-six subjects were recruited and allocated randomly (1:1:1) into three study groups. Compared to the waitlist group, both treatment groups demonstrated statistically significant decreases in TNSS and RQLQ at the end of treatment as well as after follow-up period (all *P *< 0.01). However, there was no statistically differences between these two treatment groups. There was no difference in the change of total IgE levels among study groups before or after the treatment. Only one patient reported adverse effects with herbal moxibustion treatment, and no adverse effects were found in others.

**Conclusions:**

This study supports that acupuncture could help relieve AR symptoms, but no evidence on additional treatment effect of herbal moxibustion was found.

*Trial registration* ChiCTR-INR-16010047 registered on November 25, 2016.

## Background

Allergic rhinitis (AR) is the inflammation of the nasal mucous membranes, which is caused by immunoglobulin E (IgE)-mediated allergic reactions to aeroallergens [1]. Its symptoms include sudden and recurrent nasal congestion, itchiness, sneezing and runny nose with thin nasal discharge [2]. It was estimated that 10–20% of the world’s population has allergic rhinitis, and the number of patients is increasing [3]. However, allergic rhinitis does not draw enough awareness, and is often misdiagnosed and mistreated [4]. Without appropriate and timely treatment, 10–39% of patients would develop bronchial asthma, or even pulmonary heart diseases [5]. The relapse rate is high and the patient’s daily life, sleep, and work may be seriously affected [6]. Current prevention and treatment approaches for AR include allergen prevention, medication, immunisation, and surgical treatment if necessary [7]. Commonly used medications for mitigating AR symptoms include corticosteroids, antihistamines, mast cell stabilizer, anticholinergics, and nasal decongestant drugs [8]. However, the effect of these medications generally only lasts for a short period and often with unwanted side effects [9, 10]. Although immunisation could change the natural progress of AR by regulating the immunity mechanism, the treatment cycle is long and expensive [11, 12]. Moreover, there is a potential risk of triggering serious allergic reaction [13]. Surgery, not only traumatic and costly, is only applicable for certain complications and comorbidities of rhinitis [14]. Therefore, there is an urgent need to identify an effective treatment for AR with low relapse rate and few side effects.

Chinese medicine (CM) is a renowned medical system that has been practiced in China for many years. ‘Biqiu’, a terminology originated from the CM classical literature ‘Yellow Emperor’s Inner Classic’ [15], is a disease in CM that demonstrates a set of symptoms similar to that of AR [16]. In the view of CM, the basic pathogenesis of ‘BiQiu’ is inadequate functioning of lung ‘Qi’ caused by external or internal pathogenic factors, often companied by the ‘Qi’ insufficiency of spleen and kidney. Acupuncture and herbal moxibustion are commonly used for treating AR symptoms (i.e. ‘Biqiu’). Acupuncture may regulate the function of lung ‘Qi’, unblocking the meridians and replenishing the ‘Qi’ of spleen and kidney. Moxibustion is another CM treatment modality that places burning moxa on acupoints. Herbal moxbustion is a form of moxibustion that applies a mixture of specific herbal paste on acupoints instead of burning moxa, which is named because of the supposed similar effect to moxibustion. Since herbal moxibustion used herbs with properties of warming effect could stimulate meridians, the channel of ‘Qi’ flowing inside the body, which could complement the effect of acupuncture, and it is supposed to improve further the clinical treatment effect.

Previous clinical studies have shown that acupuncture or herbal moxibustion alone could improve AR symptoms, reduce the recurrence rate, and improve the quality of life [17–20]. A systematic review involving 13 studies has reported in 2015 that acupuncture could decrease nasal symptom scores, medication scores, and serum IgE level [18]. Acupoint herbal patching alone or combined with Western medicine was also found to be more effective than placebo or Western medicine on improving total clinical symptoms and signs and quality of life in a systematic review including 20 RCTs [19, 20]. However, the quality of some previous clinical studies was generally low, and there is still no sound evidence to establish the effect of herbal moxibustion with acupuncture on AR [21, 22]. In view of herbal moxibustion being extensively applied in the treatment of allergic rhinitis with advantages of favourable treatment effect, convenient operation, good acceptability and compliance, and few side effects [19], it is worthwhile to explore further the combination of acupuncture and herbal moxibustion for the management of AR.

Our trial expands upon the pervious findings of acupuncture for AR by: (1) including the herbal moxibustion treatment as an adjunct on acupuncture; and (2) studying patients with clinical diagnosis of AR. Thus, this trial was designed to test three hypotheses: (1) acupuncture combined with herbal moxibustion was effective on treating AR symptoms compared to waiting list; (2) acupuncture was effective on treating AR symptoms compared to waiting list; (3) acupuncture combined with herbal moxibustion was more effective than acupuncture alone on the treatment of AR symptoms.

## Methods

**Fig. 1:**
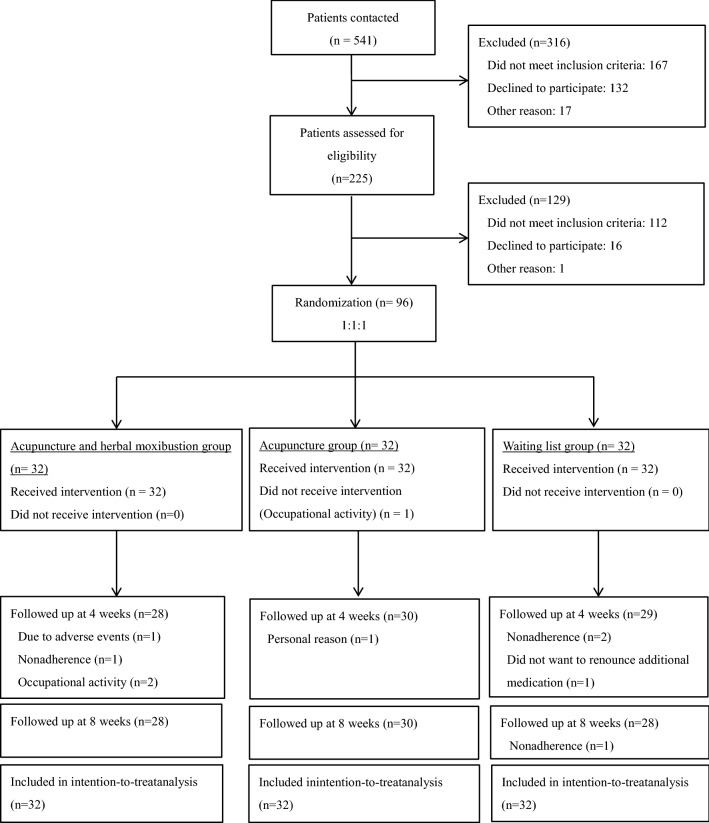
Flow diagram of overall study design and number of participants

This study was a prospective, single-centre, three-arm, assessor-blinded, randomized controlled trial. The trial participants with AR symptoms were assigned randomly to the acupuncture and moxibustion group, the acupuncture group or the waitlist group according to the ratio of 1:1:1. Overall study design was shown in Fig. 1. The Declaration of Helsinki Good Clinical Practice guidelines for trial conduct was followed throughout the trial.

### Trial participants

#### Recruitment

From September 2016 to May 2017, an open recruitment for eligible subjects was carried out in the Nethersole Chinese Medicine Service cum The Chinese University of Hong Kong Chinese Medicine Clinical Training and Research Centre. Promotional flyers were posted in the United Christian Hospital and Alice Ho Miu Ling Nethersole Hospital. Recruitment was also advertised in local newspapers, e-mails, and the internet social media (i.e. facebook of United Christian Nethersole Community Health Service).

#### Inclusion criteria

Based on the clinical diagnosis of AR in Western medicine [23], subjects were included upon meeting the following criteria:

1. Medical history of AR, which was related to change in temperature and allergens such as pollen, dust mites [8];
2. At least 2 or more of the primary symptoms of nasal congestion, runny nose, sneezing, and nasal itchiness;
3. Aged between 18–65;
4. Total Nasal Symptom Score (TNSS) of 4 or above;
5. Voluntary participation upon signing the study consent form and was fully capacitated in expressing personal will and accurately describing their disease symptoms.

#### Exclusion criteria

Subjects were excluded when they met one of the following criteria:

1. Had undertaken CM treatment related to AR or ‘BiQiu’ over the past month;
2. Had contracted respiratory infection or acute paranasal sinusitis within the past 14 days;
3. Had a history of chronic paranasal sinusitis;
4. Had organic nasal pathological changes or ever received nasal surgeries;
5. Had asthma or other respiratory attack conditions;
6. Had taken antihistamines, steroids, decongestants (used on the nose, mouth, and eyes), antibiotics, anti-coagulant, and anti-platelet agents in the past month;
7. Had undertaken specific immune treatment or systemic hormone therapy in the past year;
8. Had serious cardiac, liver, renal, cerebrovascular conditions, or neural or mental disorders;
9. Were pregnant or nursing, or planning to conceive during the course of trial;
10. Hadglucose-6-phosphate dehydrogenase deficiency (G6PD);
11. Had diabetes mellitus or wounds at the punctured locations.

#### Criteria for discontinuation

Subjects would be discontinued from the trial if one of the followings occurred:

1. The subject took prohibited drugs (antihistamines, steroids, decongestants, antibiotics, anti-coagulant and anti-platelet agents) or undertook other therapies that could affect the results of the trial;
2. The subject had sudden or serious complications over the course of treatment;
3. The subject ran into serious adverse events or allergic reactions or requested to discontinue with the trial because of adverse events; or a trial operator considered it necessary to terminate the trial because of adverse events;
4. The subject had low compliance, or failed to persist with the treatment and requested to be withdrawn;
5. Rhinitis symptoms worsen and required timely treatment, or the subject was considered unsuitable to continue with the trial according to the judgement of the research investigator.

Registered CM practitioners (CMPs) were responsible for recording whether participants had taken any prohibited drugs or received other therapies prior to each treatment session. Reasons for any discontinuance were recorded. The impact on the overall study was noted in the study report.

### Written informed consent

Written informed consent was obtained from all eligible participants. Consent form information included descriptions of the study background, detailed treatment procedures, and information on potential benefits and risks. Eligibility of participants was screened by an independent study evaluator. Consent forms were distributed to eligible participants and any questions or concerns regarding the informed consent were discussed. Voluntary signature on the consent form was obtained prior to recruitment.

### Randomization and blinding

Eligible participants were randomly allocated to one of the three study groups (i.e. the combination of acupuncture and herbal moxibustion, the acupuncture, and the waiting list group) in a 1:1:1 ratio. A random number list was generated using a computer program (Random Allocation Software) [24]. Based on the generated random number list, cards with serial numbers and allocated interventions were prepared and sealed in opaque envelopes. Only the serial number was disclosed on the envelope. Each eligible participant was allocated with a serial number. An envelope with the corresponding serial number was given to the CMP, who then provided the intervention as indicated on the card inside the envelope. Assessor-blinding was strictly implemented. The assessors were kept from knowing the treatment assignment, and did not involve any treatment during the study.

### Interventions

This study consisted of 3 study groups: acupuncture, acupuncture plus herbal moxibustion, and waitlist group. For all 3 groups, same advice on healthy lifestyle was provided by CMPs. An information sheet on the advised lifestyle, exercise, diet, and warnings to avoid possible allergens was designed and given to the patients. Explanations were also provided face-to-face.

Two registered CMPs were responsible for treatment administration. All registered CMPs received 5 years undergraduate CM education and at least 2 years of clinical experience on acupuncture treatment. To ensure consistency of treatments, training on the process of research and the manipulation of acupuncture and herbal moxibustion treatment was provided.

The patients in the acupuncture and herbal moxibustion treatment group received the acupuncture treatment first, then the herbal moxibustion treatment, and health advice during the study. The patients in the acupuncture group received only the same acupuncture treatment and health advice. Those in the waitlist group only received the health advice. Details of the study interventions were described below.

#### Acupuncture treatment

Following CM principles for treatment, acupoints LI20 (Yingxiang), GB20 (Fengchi), EX-HN3 (Yintang), LI4 (Hegu), and ST36 (Zusanli) were used in this study (Table 1). ‘Hua Tuo brand’ disposable sterile acupuncture filiform needles of the 0.25 × 25 mm or 0.25 × 40 mm specification were used. All sites of acupuncture points were sterilised with 75% alcohol pad (Bolikim Brand).Needles were inserted using the lifting-thrusting and twirling-rotating techniques until ‘deqi’ [25] sensation was achieved. The needles remain inserted for 20 min for each session. The acupuncture treatment was conducted 3 times per week for 4 weeks (a total of 12 sessions in 1 month).

**Table 1:** Summary of acupoint locations

Acupoints	Location	Needling depth	Treatment method
Yingxiang (LI20)	At the midpoint lateral to the border of the ala nasi, in the nasolabial groove	0.2–0.5 in.	Diagonal needling to de qi
Fengchi (GB20)	Below the occiput, approximately midway between Fengfu (DU16) and Wangu (GB12), in the hollow between the origins of the sternomastoid and trapezius muscles	0.5–1 in.	Diagonal needling to de qi
Hegu (LI4)	On the dorsum of the hand, between the 1st and 2nd metacarpal bones, in the middle of the 2nd metacarpal bone on the radial side	0.5–1 in.	Vertical needling to de qi
Zusanli (ST36)	Below the knee, 3 cun inferior to Dubi (ST35), one fingerbreadth lateral to the anterior crest of the tibia	0.8–1.2 in.	Vertical needling to de qi
Yintang (EX-HN3)	At the forehead, at the midpoint between the two medial ends of the eyebrow		Herbal moxibustion
Dazhui (GV14)	On the midline at the base of the neck, in the depression below the spinous process of the seventh cervical vertebra		Herbal moxibustion
Feishu (BL13)	On the back, 1.5 cun lateral to the lower border of the spinous process of the 3rd thoracic vertebra		Herbal moxibustion
Shenshu (BL23)	On the back, 1.5 cun lateral to the lower border of the spinous process of the 2nd lumbar vertebra		Herbal moxibustion

#### Herbal moxibustion treatment

‘Tianjiu’ No. 1 (for external use) designed by the Hong Kong Hospital Authority Chinese Medicine Service Department was used. Its composition was listed in Table 2. The herbal paste used in moxibustion was prepared by mixing 240 ml of ginger juice and 200 g of powdered medicine (‘Tianjiu’ No. 1). The paste was then cut into circular pellets weighing 2–3 g each. Each pellet had a surface area of 10 mm diameter and about 4–5 mm thick. A 4 cm × 4 cm plaster was used to fix the medicine onto the following acupuncture points: GV14 (Dazhui), BL13 (Feishu), and BL23 (Shenshu) (Table 1). After mounting the herbal patch, the skin might experience a mild itchiness and burning sensation. The patch was removed after 1 h. Herbal patches were applied once a week for 4 weeks. Notes on caring for the medicine application areas were provided to the participants.

**Table 2:** Composition of powdered medicine ‘Tianjiu’ No.1

Ingredient	Gram (per 200 g of ‘Tianjiu’ No. 1)
Sinapis Semen (Jiezi)	44.4 g
CorydalisRhizoma (Yanhusuo)	22.2 g
Kansui Radix (Gansui)	22.2 g
AsarumSieboldiiMiq (Xixin)	22.2 g
EphedraeHerba (Mahuang)	22.2 g
AconmLateralis RadixPraeparaia (Fuzi)	22.2 g
Cinnamomi Cortex (Rougui)	22.2 g
CaryophylliFlos (Dingxiang)	22.2 g

#### Follow-up visits

Follow-up visits were conducted within 1 month upon treatment completion. No treatment targeting AR symptoms (‘Biqiu’) were provided during this period.

### Outcome measures

#### Primary outcome

Total Nasal Symptom Score (TNSS), which is the sum of individual scores for each symptom evaluated by patients [26]. The symptoms include nasal congestion, runny nose, nasal itching and sneezing. Each symptom is to be ranked on a scale of five, according to their level of seriousness, i.e. no symptoms (0), mild (1), moderate (2), heavy (3) and severe (4).

#### Secondary outcomes

1. The Rhinoconjunctivitis Quality of Life Questionnaire (RQLQ), which has 28 questions in 7 domains (activity limitation, sleep problems, nose symptoms, eye symptoms, non-nose/eye symptoms, practical problems and emotional function) [27]. Each symptom is to be ranked on a scale of seven, according to their level of seriousness, i.e. not impaired at all (0), almost not impaired (1), slightly impaired (2), moderately impaired (3), quite impaired (4), very impaired (5), extremely impaired (6).
2. Total IgE in serum (kU/L) test (Access Total IgE Reagent Pack Cat. No. 35000) was measured in the United Christian Nethersole Community Health Service Pathology Laboratory.

All the outcome measurements were collected before the initiation of treatment, 4 weeks after treatment commencement and 1 month after the end of treatment. In addition to the above outcomes, demographic data of subjects were collected at baseline.

#### Safety assessment

Any adverse events were monitored by registered CMPs after each treatment, who examined any possible adverse conditions such as pain, haemorrhage, needle faintness, or needle sticking. Any possible redness, heatedness, itchiness, pain or even blisters appearing on the skin after herbal moxibustion was also monitored and recorded. Occurrence and severity of adverse events, as well as the handling method, were documented in the adverse events (reaction) report form. Any serious adverse events would be reported to corresponding ethical committee.

### Sample size calculation

Based on a previous study [28], we presumed that the standard deviation of TNSS is 3.6, and the mean difference of TNSS score between the acupuncture group and the waiting list group is 1.8. We firstly calculated a sample size (N) based on independent t test with a power of 80% and 5% false positive error). An estimated Pearson correlation coefficient (R) of 0.76 was made based on a previous study [29]. Sample size required for the current trial, considering ANCOVA for subsequent statistical analysis, was computed using a formula proposed by George F. Borm [30]. It was estimated that 27 subjects would be required per study group. Considering the withdrawal rate of 20%, the sample size was 32 per group (a total of 96 subjects). The computer program PASS12.0 (NCSS LLC) was used for the calculation.

### Statistical analysis

Computer program IBM SPSS Statistics 23 (IBM Analytics, US) was used for the data analysis. Descriptive statistics were calculated for all the baseline variables and outcome measures. Differences in baseline parameters among the three groups were analysed by One-way ANOVA test, Pearson X^2^ test, or Welch’s ANOVA correspondingly. The effectiveness of the interventions on all outcomes was evaluated among the three groups using generalized estimating equations (GEE) [31] due to the correlated structure of data from repeated measures at baseline, post-treatment, and 1-month follow-up. The GEE methodology was used to estimate the regression coefficient and variance of the regression coefficient when correlated data are used in regression analyses. For all GEE analyses an unstructured correlation structure was used. For missing data, maximum-likelihood estimation was used in the GEE methodology, providing estimates for the model’s parameters by finding particular parametric values that renders the observed results most probable. For cases with missing data, baseline observation carried forward was used for sensitivity analysis, and also as-treated analysis.

## Results

Between December 2016 and August 2017, 541 patients with AR symptoms (as diagnosed with ‘Biqiu’ in CM clinic) were contacted by telephone, 225 were assessed for eligibility, and finally 96 patients with 32 in each group were enrolled in the study (Fig. 1). Eighty-six of 96 patients (88.5%) (28 in acupuncture and herbal moxibustion group, 30 in acupuncture group, and 27 in waiting list group) completed the study. Ten patients were lost to follow-up (4 in acupuncture and herbal moxibustion group, 2 in acupuncture group, and 4 in waiting list group). Personal reasons, including occupational activity and non-adherence were noted for dropping out of trial.

### Baseline characteristics

Baseline demographic characteristics and clinical data of the three groups were comparable at baseline except the gender (Table 3). There were 16 (50%), 26 (81.3%) and 20 (62.5%) females in the acupuncture combing herbal moxibustion group, acupuncture, and waiting list group, respectively. And there were 16 (50%), 6 (18.7%) and 12 (37.5%) males in the acupuncture combing herbal moxibustion group, acupuncture, and waiting list group, respectively.

**Table 3:** Comparison of baseline characteristics between study groups

	Acupuncture and herbal moxibustion (N = 32)	Acupuncture (N = 32)	Waitlist (N = 32)	*P* value
Age (years)	43.59 ± 13.67	44.41 ± 13.63	41.81 ± 14.80	0.752^a^
Female, N (%)	16 (50%)	26 (81.3%)	20 (62.5%)	0.031^b^
Disease duration (years)	19.75 ± 12.62	16.22 ± 10.43	17.31 ± 12.01	0.47^a^
TNSS score	9.25 ± 2.98	9.03 ± 2.83	8.34 ± 2.99	0.439^a^
Overall RQLQ score	3.10 ± 0.87	2.94 ± 0.98	2.73 ± 0.95	0.285^a^
Individual 7 domain scores				
Daily activities	4.21 ± 0.94	4.08 ± 1.11	3.81 ± 1.27	0.352^a^
Sleep	2.76 ± 1.50	2.84 ± 1.51	2.43 ± 1.34	0.481^a^
Non-hay fever symptoms	3.13 ± 1.12	2.79 ± 1.19	2.76 ± 1.31	0.410^a^
Practical problems	4.14 ± 1.46	3.97 ± 1.46	3.49 ± 1.69	0.225^a^
Nasal symptoms	3.73 ± 1.15	3.39 ± 1.27	3.31 ± 1.32	0.359^a^
Eye symptoms	2.27 ± 1.12	1.88 ± 1.29	1.66 ± 1.28	0.141^a^
Emotion	1.91 ± 1.33	2.23 ± 1.41	2.02 ± 1.25	0.607^a^
Total IgE (kU/L)	157.16 ± 152.3	145.53 ± 223.18	268.78 ± 452.56	0.381^c^

All data presented as mean ± standard deviation unless otherwise stated

^a^One-way ANOVA test

^b^Pearson X^2^ test

^c^Welch’s ANOVA

According to CM syndrome differentiation by CMPs, recruited subjects were found to exhibit ‘BiQiu’ disease involving the lung (75 subjects), spleen (34 subjects), kidney (15 subjects), and other organ systems (13 subjects).

### Treatment effect

**Fig. 2:**
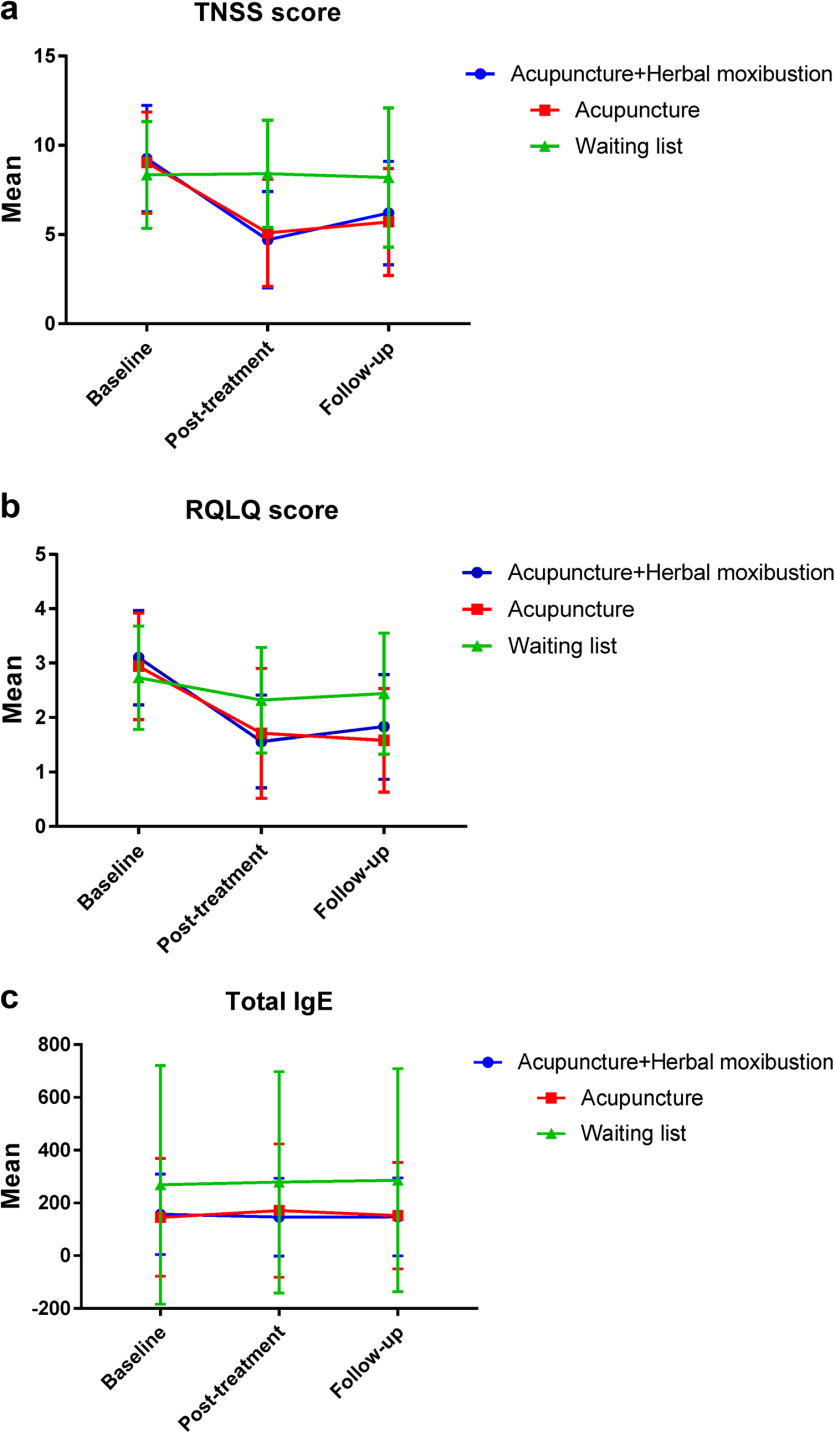
TNSS, RQLQ and total IgE during the study among three groups

Both the acupuncture plus herbal moxibustion group and acupuncture group demonstrated significant decreases in TNSS over time. Acupuncture combining herbal moxibustion and acupuncture alone decreased TNSS (mean − 4.6, 95% CI [− 6.1 ~ − 3.1], *P *< 0.001 for acupuncture and herbal moxibustion group; mean − 4.0[− 5.4 ~ − 2.5], *P *< 0.001 for acupuncture group) when compared to the waiting list at the end of treatment (Table 4). Although acupuncture plus herbal moxibustion showed a slightly larger decrease in TNSS scores than that of acupuncture group at the end of treatment period, the difference was not statistically significant (*P *= 0.42) (Fig. 2a). Sensitive analysis using baseline observation carry forward (BOCF) and as-treated analysis showed similar results (Table 4).

**Table 4:** Primary and secondary outcomes

Primary analysis													
	Acupuncture and herbal moxibustion (N = 32)		Acupuncture (N = 32)		Waiting list (N = 32)		Acupuncture and herbal moxibustion vs. acupuncture		Acupuncture and herbal moxibustion vs. waiting list		Acupuncture vs. waiting list		*P*-value^+^
	n mean (SD)		n mean (SD)		n mean (SD)		β (95% CI)	*P*-value*	β (95% CI)	*P*-value*	β (95% CI)	*P*-value*	
TNSS score													< 0.01
Weeks 4	29	4.7 (2.7)	30	5.1 (3.0)	29	8.4 (3.0)	− 0.6 (− 2.1 ~ 0.9)	0.42	− 4.6 (− 6.1 ~ − 3.1)	< 0.001	− 4.0 (− 5.4 ~ − 2.5)	< 0.001	
Weeks 8	29	6.2 (2.9)	30	5.7 (3.0)	28	8.2 (3.9)	0.4 (− 1.3 ~ 2.1)	0.67	− 2.8 (− 4.6 ~ − 1.1)	0.002	− 3.2 (− 5.0 ~ − 1.4)	< 0.001	
RQLQ score													< 0.01
Weeks 4	29	1.56 (0.85)	30	1.71 (1.19)	29	2.32 (0.97)	− 0.3 (− 0.8 ~ 0.2)	0.22	− 1.1 (− 0.7 ~ − 1.6)	< 0.001	− 0.8 (− 0.4 ~ − 1.3)	0.001	
Weeks 8	29	1.83 (0.96)	30	1.58 (0.95)	28	2.44 (1.11)	0.1 (− 0.4 ~ 0.61.56)	0.65	− 1.0 (− 0.6 ~ − 1.4)	< 0.001	− 1.1 (− 0.6 ~ − 1.6)	< 0.001	
Total IgE													0.14
Weeks 4	29	146.4 (147.5)	30	171.3 (252.4)	29	278.6 (419.7)	− 19.3 (− 41.8 ~ 3.3)	0.09	12.3 (− 21.0 ~ 45.6)	0.47	31.5 (− 3.9 ~ 67.0)	0.08	
Weeks 8	29	147.2 (147.7)	29	152.0 (201.7)	28	286.5 (422.5)	5.9 (− 19.1 ~ 31.0)	0.64	8.9 (− 33.2 ~ 51.0)	0.68	2.9 (− 41.7 ~ 47.5)	0.90	
Sensitivity analysis (primary outcome)													
BOCF imputation													< 0.01
Weeks 4	32	5.1 (2.9)	32	5.3 (2.9)	32	8.3 (3.0)	− 0.4 (− 1.9 ~ 1.1)	0.62	− 4.1 (− 5.5 ~ − 2.7)	< 0.001	− 3.7 (− 5.1 ~ − 2.3)	< 0.001	
Weeks 8	32	6.5 (3.0)	32	5.8 (2.9)	32	8.2 (3.8)	0.5 (− 1.2 ~ 2.2)	0.56	− 2.7 (− 4.3 ~ − 1.1)	0.001	− 3.2 (− 4.8 ~ − 1.5)	< 0.001	
As treated													< 0.01
Weeks 4	29	4.7 (2.7)	30	5.1 (3.0)	29	8.4 (3.0)	− 0.7 (− 2.2 ~ 0.9)	0.40	− 4.8 (− 6.2 ~ − 3.4)	< 0.001	− 4.1 (− 5.5 ~ − 2.8)	< 0.001	
Weeks 8	29	6.2 (2.9)	30	5.7 (3.0)	28	8.2 (3.9)	0.4 (− 1.4 ~ 2.2)	0.65	− 2.9 (− 4.7 ~ − 1.2)	0.001	− 3.3 (− 5.2 ~ − 1.5)	< 0.001	

*GEE model, comparing change from baseline between groups, model: intercept, group, visit, gender, group by visit interaction effect

^+^GEE model, *P*-value of group by visit interaction effect

Compared to waiting list group at the end of follow-up period, acupuncture plus herbal moxibustion and acupuncture alone also decreased TNSS (mean − 2.8, 95% CI [− 4.6 ~ − 1.1], *P *< 0.01for acupuncture and herbal moxibustion group; mean − 3.2, 95% CI [− 5.0 ~ − 1.4], *P *< 0.001 for acupuncture group). However, the difference between the two active treatment groups was not statistically significant (*P *= 0.67).

In addition to TNSS, RQLQ was also found to be significantly reduced in both the acupuncture plus herbal moxibustion treatment group and acupuncture treatment group when compared to the waiting list group (mean − 1.1, 95% CI [− 0.7 ~ − 1.6], *P *< 0.001 for acupuncture and herbal moxibustion group; mean − 0.8, 95% CI [− 0.4 ~ − 1.3], *P *< 0.01 for acupuncture group) (Fig. 2b). Statistically significant reduced RQLQ was also found at the end of follow-up period for the two treatment groups when compared to the waitlist group (mean − 1.0, 95% CI [− 0.6 ~ − 1.4], *P *< 0.001 for acupuncture and herbal moxibustion group; mean − 1.1, 95% CI [− 0.6 ~ − 1.6], *P *< 0.001 for acupuncture group) (Table 4).

In addition to Overall RQLQ score, the domain scores of RQLQ were also found to be reduced in both the acupuncture plus herbal moxibustion treatment group and acupuncture treatment group when compared to the waiting list group (P < 0.05), except in the domains of practical problems between acupuncture group and waiting list group after treatment (P = 0.095) (Table 5). Statistically significant reduction in the domain scores of RQLQ was also found at the end of follow-up period for the two treatment groups when compared to the waitlist group (P < 0.05) (Table 5).

**Table 5:** Secondary Outcomes (RQLQ score)

	Acupuncture and herbal moxibustion (N = 32)		Acupuncture (N = 32)		Waiting list (N = 32)		Acupuncture and herbal moxibustion vs. acupuncture		Acupuncture and herbal moxibustion vs. waiting list		Acupuncture vs. waiting list		*P* value^+^
	n mean (SD)		n mean (SD)		n mean (SD)		β (95% CI)	P value*	β (95% CI)	P value*	β (95% CI)	P value*	
Daily activities													< 0.001
Weeks 4	29	2.7 (1.1)	30	2.7 (1.3)	29	3.9 (1.2)	− 0.1 (− 0.8 ~ 0.6)	0.775	− 1.6 (− 2.1 ~ − 1.0)	< 0.001	− 1.5 (− 2.1 ~ − 0.9)	< 0.001	
Weeks 8	29	3.1 (1.0)	30	2.9 (1.4)	28	3.9 (1.0)	0.1 (− 0.5 ~ 0.8)	0.728	− 1.2 (− 1.7 ~ − 0.7)	< 0.001	− 1.3 (− 2.0 ~ − 0.6)	< 0.001	
Sleep													0.002
Weeks 4	29	1.4 (1.1)	30	1.4 (1.4)	29	2.1 (1.4)	0.1 (− 0.7 ~ 0.8)	0.858	− 1.1 (− 1.7 ~ − 0.4)	0.001	− 1.1 (− 1.9 ~ − 0.4)	0.003	
Weeks 8	29	1.5 (1.4)	30	1.2 (1.3)	28	2.2 (1.6)	0.5 (− 0.3 ~ 1.3)	0.213	− 1.0 (− 1.7 ~ − 0.2)	0.010	− 1.5 (− 2.3 ~ − 0.6)	< 0.001	
Non-hay fever symptoms													< 0.001
Weeks 4	29	1.5 (1.1)	30	1.7 (1.3)	29	2.3 (1.0)	− 0.5 (− 1.1 ~ 0.1)	0.130	− 1.2 (− 1.7 ~ − 0.6)	< 0.001	− 0.7 (− 1.3 ~ − 0.1)	0.021	
Weeks 8	29	1.7 (1.3)	29	1.5 (1.1)	28	2.3 (1.3)	− 0.1 (− 0.7 ~ 0.4)	0.702	− 1.0 (− 1.6 ~ − 0.5)	< 0.001	− 0.9 (− 1.6 ~ − 0.3)	0.003	
Practical problems													0.003
Weeks 4	32	1.9 (1.3)	32	2.1 (1.7)	32	2.3 (1.4)	− 0.3 (− 1.2 ~ 0.6)	0.539	− 1.0 (− 1.9 ~ − 0.2)	0.018	− 0.7 (− 1.6 ~ 0.1)	0.095	
Weeks 8	29	2.1 (1.4)	30	1.9 (1.3)	28	2.7 (1.7)	0.1 (− 0.6 ~ 0.9)	0.754	− 1.2 (− 1.9 ~ − 0.4)	0.003	− 1.3 (− 2.0 ~ − 0.5)	0.001	
Nasal symptoms													0.003
Weeks 4	29	1.9 (0.9)	30	2.1 (1.3)	29	2.7 (1.1)	− 0.6 (− 1.2 ~ 0.1)	0.082	− 1.3 (− 1.9 ~ − 0.6)	< 0.001	− 0.7 (− 1.3 ~ − 0.1)	0.033	
Weeks 8	29	2.2 (1.1)	30	2.1 (1.3)	28	2.8 (1.3)	− 0.2 (− 0.8 ~ 0.4)	0.482	− 1.0 (− 1.6 ~ − 0.4)	0.002	− 0.8 (− 1.4 ~ − 0.1)	0.017	
Eye symptoms													< 0.001
Weeks 4	29	1.0 (1.0)	30	1.1 (1.4)	29	1.4 (1.4)	− 0.4 (− 0.9 ~ 0.1)	0.135	− 1.0 (− 1.6 ~ − 0.4)	0.001	− 0.6 (− 1.2 ~ − 0.1)	0.024	
Weeks 8	29	1.5 (1.1)	30	0.9 (1.0)	28	1.7 (1.4)	0.3 (− 0.3 ~ 0.8)	0.346	− 0.9 (− 1.4 ~ − 0.3)	0.002	− 1.1 (− 1.7 ~ − 0.6)	< 0.001	
Emotion													0.001
Weeks 4	29	0.8 (0.9)	30	1.2 (1.3)	29	1.8 (1.4)	− 0.1 (− 0.7 ~ 0.4)	0.648	− 0.9 (− 1.5 ~ − 0.3)	0.003	− 0.8 (− 1.4 ~ − 0.2)	0.011	
Weeks 8	29	1.0 (1.2)	30	1.0 (1.1)	28	1.9 (1.4)	0.3 (− 0.3 ~ 0.9)	0.294	− 0.8 (− 1.4 ~ − 0.2)	0.006	− 1.2 (− 1.8 ~ − 0.6)	< 0.001	

*GEE model, comparing change from baseline between groups, model: intercept, group, visit, gender, group by visit interaction effect

^+^GEE model, p-value of group by visit interaction effect

There was no significant difference in total IgE among the 3 study groups after treatment or one-month follow-up period (Fig. 2c and Table 4).

In this study, among the 96 patients, only one patient in the acupuncture and herbal moxibustion group developed adverse effects with herbal moxibustion. No adverse effects were reported by patients in the acupuncture group and waiting list group.

## Discussion

This study demonstrated a beneficial effect of acupuncture with or without moxibustion on relieving the main clinical symptoms and improving the quality of life in patients with AR symptoms (‘BiQiu’). No additional benefit was found when herbal moxibustion was used with acupuncture in this study. To our knowledge, this study was the first clinical trial in Hong Kong that evaluated the clinical effectiveness of acupuncture with or without herbal moxibustion on relieving AR symptoms (‘BiQiu’).

‘BiQiu’ is a nasal disease characterised by sudden and recurring nasal itchiness, sneezing, runny nose with thin nasal discharge, nasal congestion. The condition can be perennial or seasonal [32]. We choose those patients only with clinical diagnosis of AR (‘BiQiu’) as this is the most commonly treatment approach in the clinical practice of Chinese medicine in Hong Kong. Generally clinical acupuncture or herbal moxibustion treatment is administered after clinical diagnosis is made not without the measure of serum specific IgE. The study findings on those with clinical diagnosis of AR (‘BiQiu’) is supposed to be of more practical value for clinicians. Because the pathogenesis of perennial or seasonal AR is different, the effect of acupuncture and herbal moxibustion is supposed to be different on them. It is valuable to explore further the effect of acupuncture on different types of AR.

In the view of Chinese medicine, the basic pathogenesis of ‘BiQiu’ is inadequate functioning of lung ‘Qi’ caused by external or internal pathogenic factors, often companied by the ‘Qi’ insufficiency of spleen and kidney. Therefore, the general CM treatment principle for ‘BiQiu’ is to regulate the function of lung ‘Qi’, unblocking the meridians and replenishing the ‘Qi’ of spleen and kidney. In the fixed acupuncture treatment regimen, LI20 (Yingxiang), GB20 (Fengchi) and EX-HN3 (Yintang) mainly could regulate the function of lung ‘Qi’ and unblock the meridians; LI4 (Hegu) and ST36 (Zusanli) mainly could replenish the ‘Qi’ of spleen. Since herbal moxibustion used herbs with properties of warming effect could stimulate meridians, the channel of ‘Qi’ flowing inside the body, they may exert the treatment effect similar to moxibustion. Such warming effect of herbal moxibustion may complement acupuncture, and it is supposed to improve further the clinical treatment effectiveness.

However, little added benefits were observed for the extra herbal moxibustion treatment. Several reasons might explain for such a lack of effect. Firstly, patients were treated with standardised treatment regime without CM syndrome differentiation [33]. According to the CM theory, herbal moxibustion could dispel cold stagnation (yin), and it may exert more treatment effect for those with syndrome of cold stagnation and qi deficiency. We have collected information about the diagnosis of syndrome differentiation made by CMPs which was made based on their usual clinical practice to make a preliminary exploration. No pre-defined standard was set for the syndrome differentiation for such an exploration on clinical practice. Thus, only the diseased organs were analysed. The individual syndrome differentiations were varied so much that it is difficult to categorize them. It was found that 55% of patients were diagnosed involving the malfunction of lung. Spleen, kidney and other organs were also involved. Secondly, the treatment time, duration and frequency of herbal moxibustion might not be adequate. In this study, herbal moxibustion was applied to patients once a week, each time not more than an hour, and a total four times during 1 month treatment. Longer therapy of over 6 weeks had ever demonstrated better effect in previous studies [34]. Moreover, herbal moxibustion is considered to take optimal effect when administered on the hottest days during the year [22]. In the CM theory, Yang Qi in the natural world at that time may help enhance the treatment effect of herbal moxibustion.

Because elevated total IgE is associated with an increased risk of allergic disease, we measured the change of IgE in the patients with AR symptoms. Some previous studies demonstrated that acupuncture and herbal moxibustion may adjust the immune function of the human body so as to lower the total IgE level [35, 36]. In this study, there was no significant difference in total IgE among the 3 study groups after treatment or one-month follow-up period. Elevations in total serum IgE are seen in several types of disorders, including allergic diseases, some primary immunodeficiencies, parasitic and viral infections, certain inflammatory diseases, some malignancies, and several other diseases. Thus, elevated total serum IgE is not specific to allergic disease. On the other side, those with low or normal serum IgE levels could still have local production of allergen-specific IgE in the tissues or a high ratio of allergen-specific to total IgE [37]. A high total IgE level alone is of limited value as a marker of allergy as it does not give any clue to sensitizing allergens in an individual. Further research on allergen-specific IgE following the acupuncture treatment for AR may be needed.

In this study, all subjects received health education on lifestyle, encouragement to carry out proper physical activities, having adequate sleep, refraining from cold food and avoiding to contact allergens, which was generally a part of ordinary CM clinical practice. From the view of CM, the onset of ‘BiQiu’ is highly related to lifestyle, which may affect the treatment effectiveness if the modification of life style is neglected. Thus, CMPs always provide advices on healthy lifestyle in order to facilitate the treatment. In addition, it would foster the compliance of the participants in the waiting list.

There were some limitations with the study. Firstly, the acupuncture treatment duration and follow-up period were relatively short for such a chronic disease. Secondly, treatment based on TCM syndrome differentiation was not used as in clinical practice, which is considered to enhance clinical effectiveness according to TCM theory. Thirdly, the treatment duration and frequency of herbal moxibustion may be not appropriate for taking effect.

## Conclusions

This study demonstrated the beneficial clinical effect of acupuncture, with or without herbal moxibustion, on symptom reduction and quality of life improvement for the AR patients. No additional beneficial effect of herbal moxibustion was found in this study.

## Data Availability

The datasets used and/or analyzed during the current study would be available from the corresponding author on reasonable request after study completion.

## Abbreviations

CM: Chinese medicine
CMP: Chinese medicine practitioner
TNSS: total nasal symptom score
G6PD: glucose-6-phosphate dehydrogenase
PASS: power analysis and sample size
SD: standard deviation
ANCOVA: analysis of co-variance
RQLQ: Rhinoconjunctivitis Quality of Life Questionnaire
IL-4: interleukin-4
IL-5: interleukin-5
